# Water Stress and Foliar Boron Application Altered Cell Wall Boron and Seed Nutrition in Near-Isogenic Cotton Lines Expressing Fuzzy and Fuzzless Seed Phenotypes

**DOI:** 10.1371/journal.pone.0130759

**Published:** 2015-06-22

**Authors:** Nacer Bellaloui, Rickie B. Turley, Salliana R. Stetina

**Affiliations:** Crop Genetics Research Unit, United States Department of Agriculture, Agricultural Research Service, Stoneville, Mississippi, United States of America; New Mexico State University, UNITED STATES

## Abstract

Our previous research, conducted under well-watered conditions without fertilizer application, showed that fuzziness cottonseed trait resulted in cottonseed nutrition differences between fuzzy (*F)* and fuzzless (*N*) cottonseed. Under water stress conditions, B mobility is further limited, inhibiting B movement within the plant, affecting seed nutrition (quality). Therefore, we hypothesized that both foliar B and water stress can affect B mobility, altering cottonseed protein, oil, and mineral nutrition. The objective of the current research was to evaluate the effects of the fuzziness seed trait on boron (B) and seed nutrition under water stress and foliar B application using near-isogenic cotton lines (NILs) grown in a repeated greenhouse experiment. Plants were grown under-well watered conditions (The soil water potential was kept between -15 to -20 kPa, considered field capacity) and water stress conditions (soil water potential between -100 and -150 kPa, stressed conditions). Foliar B was applied at a rate of 1.8 kg B ha^-1^ as H_3_BO_3_. Under well-watered conditions without B the concentrations of seed oil in *N* lines were higher than in *F* lines, and seed K and N levels were lower in *N* lines than in *F *lines. Concentrations of K, N, and B in leaves were higher in *N *lines than in *F *lines, opposing the trend in seeds. Water-stress resulted in higher seed protein concentrations, and the contribution of cell wall (structural) B to the total B exceeded 90%, supporting the structural role of B in plants. Foliar B application under well-watered conditions resulted in higher seed protein, oil, C, N, and B in only some lines. This research showed that cottonseed nutrition differences can occur due to seed fuzziness trait, and water stress and foliar B application can alter cottonseed nutrition.

## Introduction

Upland cotton (*Gossypium hirsutum* L.) is a major crop in the world because of its natural fiber and cottonseed composition (protein, oil, fatty acids, and mineral nutrition). Cottonseed is a source of oil for human consumption [1– 3] and cottonseed meal for livestock [1, 4, 5]. Cottonseed oil production ranks third after soybean and rapeseed [6]. Protein in cottonseed ranges from 17–27% and oil from 12–30%, saturated fatty acids ~29%, and unsaturated fatty acids ~70% [2, 3, 5, 7]. Cottonseed oil has a desirable trait in that it contains enough saturated fatty acids (~ 22% palmitic, ~3% stearic, ~ 1% myristic acid) to prevent partial hydrogenation, making it relatively stable. Also, it contains sufficient unsaturated fatty acid, making it beneficial for human health (22% oleic acid, ~52% linoleic acid, and <1% linolenic acid). Although seed composition constituents are genetically controlled, they are also found to be influenced by environmental factors such growing seasons, locations, and years [4, 8], temperature [9], and agricultural practices [10–12]. Therefore, maintaining high quality of cottonseed nutrition under different environments is essential.

The genetic basis controlling protein and oil in fuzzless cottonseed has received little attention [5], and understanding the genotypic and environmental factors influencing cottonseed composition may result in a better product [2, 3]. Protein, oil, and fatty acids were investigated in several conventional cotton genotypes in 2006 and 2007 in a total of nine environments in six locations, and found that the level of most fatty acids was influenced by environment and genotype, but the interaction between environment and genotype was relatively small [2]. A comparative study was conducted on fatty acids among *G*. *hirsutum* genotypes and hybrids and concluded that the fatty acids in cottonseed lines significantly differ and the degree of variability of fatty acids was influenced by the conditions during the period of the formation and ripening of the seeds, and these researchers were able to recommend lines as donors to improve the food-value indices of cottonseed oil [13]. They also observed that higher levels of saturated fatty acids occurred in the second year, with lower average humidity and higher temperature compared with the conditions of the first year [13]. This observation was in agreement with those found by others [2] in that the higher temperatures and drier growing conditions reduced linoleic acid and increased saturated fatty acids, and this response of fatty acids was dependent on genotype. It was also found that there was a significant effect of genotype × environment interactions, which did not agree with the findings of some other researchers [2]. The effects of planting dates on seed nutrition (protein, oil, fatty acids, carbohydrates, and gossypol) were investigated using six conventional cotton cultivars planted late April and late May under irrigated and non-irrigated field conditions near Stoneville, MS, USA in 2005 through 2008 [3]. They found that irrigation resulted in higher oil and total soluble carbohydrates in seeds, and lower protein and saturated fatty acids content. Also, they found that the effect of late April planting on the fatty acid distribution was similar to that observed under dryland conditions. They concluded that, depending on the desirable benefits, growers can adopt production strategies to produce cottonseed with desirable compositional qualities. It was also reported that variability in seed fatty acid composition exists among cotton genotypes and cultivars [2, 3, 13, 14], indicating the possibility for improved seed composition quality [3]. It was suggested that alteration or optimization of cottonseed composition may improve the cottonseed value and expand the use of its products [13].

Boron (B) is an essential micronutrient for cotton growth, development, production, and seed quality [15]. Cotton has a high B requirement, partially, due to the inherently limited B mobility within cotton plants. Although B was shown to affect yield and seed and fiber quality in cotton, its effects on seed nutrition have not been well investigated [3, 15, 16], especially under water stress [3, 16]. It was reported that a level of <20 mg kg^-1^ is considered deficient level and the range between 20 and 80 mg kg^-1^ is sufficient. However, other researchers established that critical level of B in recently matured cotton leaf blades is 53 mg B kg^-1^ [17] rather than the generally listed 15–20 mg B kg^-1^ in the literature [18, 19]. This is due to the fact that B taken up by roots of non-sugar alcohol species such as cotton is largely phloem-immobile, and B concentrations in matured fully expanded leaves may not be physiologically relevant to B status in plants at time of sampling, leading to over- or underestimation of B requirement for plant growth. Therefore, the adequacy range of B in plant tissue is not clearly defined and difficult to interpret [15]. This complexity of B status in tissue may have led to report that B can be deficient in a number of crops and have significant effect on yield [15, 16] and quality [15, 16] even when there are no vegetative signs of deficiency and even when B concentration in soil is present at the adequate range [15, 20, 21]. Environmental factors (soil moisture, soil texture, and soil reaction) [22, 23], species and cultivar [24], and cell wall composition that affect B uptake and requirement is also another source of variability determining B nutritional status of cotton plants. For example, it was found that most of the variations in B concentration among species could be attributed to variation in cell wall composition and pectin levels in cell as species containing high pectin levels in cell walls maintain greater internal B concentrations [25].

In the United States, the upland cotton is the predominant species and produces on its seeds two types of fibers, lint and fuzz [26–29]. Lint is long, faster growing, and commercially valuable fiber, and the fuzz fiber is short and slow growing; during the ginning process the fuzz fiber is left behind after the removal of fiber lint [30]. Because previous research reported that several physiological and genetic processes are involved in fuzz fiber development, including carbon metabolism and mineral assimilations [28, 31], our hypothesis was that exposing the plants to water stress and mineral foliar application may further alter the level and trend of cottonseed protein, oil, and minerals. Understanding the changes in seed nutrition under different environments is critical for trait stability and its effects on the physiological and biochemical process, especially those involved in seed nutrition. Because much remains unclear about the effects of water stress and B nutrition on cottonseed nutrition [3, 5, 32], and to avoid the confounding effects of genotype, the objective of the current study was to investigate the effects of water stress and foliar B application on cottonseed nutrition using two sets of near-isogenic cotton lines for the fuzz trait [Mississippi Delta 51*ne* (MD 51*ne F* and MD 51*ne N*) and Stoneville 7A *gl* (STV 7A*gl F* and STV 7A*gl N*)], with SA 243 (source of the dominant fuzzless allele, *N*_*1*_ *N*_*1*_), and PHY 375WRF (Dow Agrosciences, Indianapolis, IN, USA) upland variety as checks. To our knowledge this is the first report on the effect of combined effects of water stress and foliar B application on seed nutrition using near-isogenic lines for the cottonseed fuzz trait.

## Materials and Methods

A greenhouse experiment was repeated twice. Each experiment was conducted in a different greenhouse bay and was separate from the other. Two sets of near-isogenic lines (NILs) were used and each set consists of two lines that share the same genetic background, but differ in the expression of seed fuzziness (fuzzy, *F*; fuzzless, *N*). Plants were divided into two groups; one group was grown under well-watered conditions (W) and the other half was grown under water stress (WS). The water stress conditions was introduced by weighing soil in pots, and then saturated with deionized water and left to drain and weighed again to obtain the water field capacity as measured by soil water sensors inserted in pots [33]. The soil water potential was kept between -15 to -20 kPa (considered field capacity) in well-watered soil and between -100 and -150 kPa in soil of water-stressed plants. To monitor the soil water potential, measurements of soil water potential were taken daily by sensors inserted in the pots and read using Soil Moisture Meter (WaterMark Company, Inc., Wisconsin, USA). The use of soil water stress levels in this experiment was based on soil field capacity for irrigated cotton in the field and water stress conditions during drought period for field stressed cotton. Field soil was used in this experiment to mimic soil under field conditions. Although cotton is irrigated when soil water potential reaches about -50 kPa, soil water potential can reach upto -199 kPa during drought periods. So, we chose a range that falls within soil water stress under field condition.

The near-isogenic sets of cotton lines were: Mississippi Delta 51*ne* (MD 51*ne F* and MD 51*ne N*); Stoneville 7A*gl* (STV 7A*gl F* and STV 7A*gl N*), and SA 243 [(source of the dominant allele (*N1*, *N1*) (PI 528610)]. PHY 375WRF, upland variety, was used as checks. Seeds were germinated and uniform size seedlings were transplanted into 9.45 L size pots filled with field soil with the following physical and chemicals characteristics. The soil was a Dundee silt loam (fine-silty, mixed, active, thermic Typic Endoqualfs) with pH 6.3, 1.1% organic matter, a cation exchange capacity of 15 cmol/kg, and soil textural fractions of 26% sand, 56% silt, and 18% clay. The concentrations of nutrients in soil were (mg kg^-1^): P = 474, K = 1217, S = 41, Ca = 1721, Mg = 1258, Mn = 195, Na = 54, B = 1.4, Zn = 23, Fe = 6094. The concentrations (g kg^-1^) of N and C in soil were 0.6 and 8.1, respectively. Plants were grown under the following greenhouse conditions: temperature was 34°C ± 11°C during the day and 28°C ± 6°C at night with a photosynthetic photon flux density (PPFD) of about 800 (cloudy day) to 2400 (sunny day) μmol·m^-2^·s^-1^, as measured at canopy height by Quantum Meter (Spectrum Technology, Inc., Illinois, USA). The range of light intensity reflects a bright sunny or cloudy day. To avoid differences in the day-length between the two experiments, the two experiments were conducted simultaneously during the normal growing season (from April to September). Foliar B was applied at a rate of 1.8 kg B ha^-1^ as H_3_BO_3_ at vegetative stage (33 days after emergence) and also at beginning of boll development stage (97 days after emergence). Four replicates were used in each experiment. The concentrations of B in leaves were determined at day 8 from the second application at the boll development stage.

The authority responsible for the greenhouses that allowed this research activity is the United States Department of Agriculture, Agricultural Research Services (USDA-ARS), Stoneville, MS. This research activity was conducted under the authority of USDA-ARS, and this greenhouse study does require any specific permission because it is owned by USDA-ARS for routine research activities. This research did not involve endangered or protected species.

### Analysis of nutrients in soil

The concentrations of P, K, Mg, Zn, Mn, and Fe were determined using a 5-g soil: 20 ml Mehlich-1 solution and analyzed using inductively coupled plasma (ICP) spectrometry at The University of Georgia’s Soil, Plant, and Water Laboratory, Athens, GA [34]. The concentrations were measured by combusting a 0.25-g sample of soil in an oxygen atmosphere at 1350°C, converting elemental nitrogen, sulfur, and carbon into N_2_, SO_2_, and CO_2_. The gases were then passed through infrared cells and N, S, and C were determined by elemental analyzer using thermal conductivity cells (LECOCNS-2000 Elemental Analyzer, LECO Corporation, St. Joseph, MI, USA).

### Analysis of N, C, and minerals in leaves and cottonseeds

Cotton leaves were analyzed for N, C, K, and B at the beginning of boll development stage (at day 8 after the second application). Mature seeds were collected, ginned, acid-delinted, and analyzed for N, C, and K. Leaf and seed samples were ground to pass through a 1-mm sieve using a Laboratory Mill 3600 (Perten, Springfield, IL). Leaf and seed K were measured by digesting 0.5 g of dried ground tissue in HNO_3_ in a microwave digestion system. The concentrations of K were determined using inductively coupled plasma spectrometry (ICP) [34]. The concentrations of N and C in leaf and seed samples were analyzed by elemental analyzer as described previously. The concentration of B in leaves and seeds was determined as indicated in the following sections. The concentration of B in leaves and seeds was determined as indicated in the following sections.

### Analysis of total boron in leaves and seeds

The concentration of B in leaves and seeds were determined with the azomethine-H method [15, 35], and as previously detailed [33, 34]. Briefly, 1 g of a dried ground seed sample was ashed at 500°C for 8 h, and then extracted with 20 ml of 2 M HCl at 90°C for 10 minutes, and filtered. Then, 2 ml of the filtrate solution was added to 4 ml of buffer solution (containing 25% ammonium acetate, 1.5% EDTA, and 12.5% acetic acid). Four ml of azomethine-H solution, containing 0.45% azomethine-H and 1% of ascorbic acid, were freshly prepared before the analysis was added to the sample mixture. Boron concentration was determined using a Beckman Coulter DU 800 spectrophotometer (Fullerton, CA) at 420 nm [33,34].

### Analysis of cell wall (structural) boron

The concentration of cell wall B in leaves was determined [36]. Briefly, 2 g of fresh fully expanded leaves were collected from each replicate and treatment and homogenized in mortar and pestle with ice in cold water. Then, the homogenate was spun in a centrifuge at 1000 g for 10 minutes, and the residue was washed three times with 10 ml of 80% ethanol and once with10 ml of methanol:chloroform mixture (1:1, v/v). The resulting precipitate was washed with 10 ml of acetone. Then, the samples were dried and ashed for cell wall B determination as previously described.

### Analysis of cottonseed protein and oil

Mature cottonseeds were collected from each plot and analyzed for protein and oil. Briefly, approximately 25 g of seed was ground using a Laboratory Mill 3600 (Perten, Springfield, IL). Seed protein and oil were analyzed by near infrared reflectance [28,37,38] using a diode array feed analyzer AD 7200 (Perten, Springfield, IL). Calibrations were developed using Perten’s Thermo Galactic Grams PLS IQ software. The calibration equation curve was established according to AOAC methods [39,40]. Protein and oil were expressed on a seed dry matter basis [37, 41].

### Experimental design and analysis

The experiment was arranged in a split-split plot design with watering as a main plot and cotton lines as sub-plot, and foliar B application as sub-sub-plot. Plots were arranged in a randomized compete block design. The experiment was repeated twice. Four replicates were used for each treatment. Each pot with three plants was considered one replicate. Analysis of variance was conducted using Proc Mixed model in SAS [42]. Foliar boron treatment, cotton line, experiment, watering treatment, and their interactions were modeled as fixed effects. Replications and the interaction between replication, watering, and boron application were considered random effects. Random effect factors were considered variance covariance parameters and their estimated residuals were indicated in Tables 1 and 2. Residual values in Tables 1 and 2 refer to Restricted Maximum Residual Likelihood (REML), which reflects the variance of the experiment unit associated with the random parameters in the model. Means were separated by Fisher’s protected least significant difference test at the 5% level of significance using SAS [42].

**Table 1:** Effect of source of variance (F value and P significant level) on cottonseed protein, oil, K, C, N, and B in near-isogenic lines (Line) expressing fuzzy and fuzzless seed phenotypes under water stress (well-watered and water stressed plants; W) with and without foliar B application (B) in a repeated (Experiment 1 and Experiment 2; Exp) greenhouse experiment.

		Protein	Oil	K (%)	C	N	B (mg kg^-1^)
Effect	DF	F	F	F	F	F	F
**Exp**	1	29.68***	20.8**	1.56***	0.51^NS^	43.2***	20.9***
**W**	1	0.00^NS^	354***	495***	154***	163***	501***
**Exp × W**	1	4.7*	31.5***	39.2***	7.56**	2.49^NS^	11.01***
**B**	1	18.0***	105***	1.07^NS^	21.5***	111***	0.70^NS^
**Exp × B**	1	18.8***	20.8***	1.07^NS^	2.57 ^NS^	1.72^NS^	1.01^NS^
**W × B**	1	2.36^NS^	0.00^NS^	0.01^NS^	15.41***	20.5***	2.54^NS^
**Exp × W × B**	1	3.99^NS^	0.35^NS^	3.72^NS^	0.24 ^NS^	3.73*	1.37^NS^
**Line**	5	16.0***	15.1***	3.10*	9.60***	7.65***	17.2***
**Exp × Line**	5	5.53***	5.77***	2.89*	1.12 ^NS^	2.54*	3.79**
**W × Line**	5	4.16***	8.69***	3.08**	8.47***	4.24***	3.32**
**Exp × W × Line**	5	2.61*	0.30^NS^	2.38*	2.73*	0.25^NS^	1.77^NS^
**B × Line**	5	5.01***	2.81*	0.37^NS^	6.86***	1.12^NS^	1.38^NS^
**Exp × B × Line**	5	5.52***	1.77^NS^	1.06^NS^	3.85**	1.30^NS^	1.60^NS^
**W × B × Line**	5	15.6***	7.34***	1.69^NS^	7.41***	1.58^NS^	4.47***
**Exp × W × B × Line**	5	2.46*	4.00***	1.12^NS^	3.22**	1.35^NS^	2.54*
**Residual**		7.40	4.50	0.02	0.24	0.17	11.90

*Significance at *P*≤0.05

**significance at *P* ≤0.01

*** significance at *P*≤0.001

**Table 2:** Effect of source of variance (F value and P significant level) on cotton leaves K, N, and total B, cell wall B (CellWB), and cell wall B contribution to the total B (CellWB %) in near-isogenic lines (Line) expressing fuzzy and fuzzless seed phenotypes under water stress (well-watered and water stressed plants; W) with and without foliar B application (B) in a repeated (Experiment 1 and Experiment 2; Exp) greenhouse experiment.

		K^1^(%)	N(%)	Total B (mg kg^-1^)	CellWB (mg kg^-1^)	CellWB (%)
Effect	DF	F	F	F	F	F
**Exp**	1	0.36^NS^	35.1***	3.04^NS^	5.11*	0.8^NS^
**W**	1	53.4***	410***	1289***	241***	149***
**Exp × W**	1	0.39^NS^	68.2***	6.51*	5.65*	0.02^NS^
**B**	1	10.8**	0.73^NS^	46.42***	3.51^NS^	23.8***
**Exp × B**	1	0.63^NS^	2.78^NS^	0.94^NS^	1.47^NS^	0.74^NS^
**W × B**	1	1.86^NS^	0.01^NS^	61.06***	2.81^NS^	4.31*
**Exp × W × B**	1	2.48^NS^	2.08^NS^	1.14^NS^	0.01^NS^	0.59^NS^
**Line**	5	4.29***	14.4***	10.71***	12.71***	1.31^NS^
**Exp × Line**	5	0.23^NS^	2.88**	3.61**	2.77*	1.75^NS^
**W × Line**	5	4.36***	0.87^NS^	13.06***	8.2***	2.25*
**Exp × W × Line**	5	1.08^NS^	2.46*	1.72^NS^	1.05^NS^	0.9^NS^
**B × Line**	5	0.66^NS^	1.11^NS^	3.12*	4.92***	1.15^NS^
**Exp × B × Line**	5	1.33^NS^	2.32*	3.57**	0.79^NS^	0.22^NS^
**W × B × Line**	5	1.41^NS^	1.34^NS^	4.34***	8.05***	1.21^NS^
**Exp × W × B × Line**	5	1.28^NS^	0.48^NS^	1.00^NS^	1.5^NS^	0.7^NS^
**Residual**		0.47	0.17	12.6	9.4	107

*Significance at *P*≤0.05

**significance at *P* ≤0.01

*** significance at *P*≤0.001.

## Results and Discussion

ANOVA analysis (Tables 1 and 2) showed that the main effect of water was significant for oil, K, C, N, and B, indicating the influence of irrigation on these constituents. Foliar B was also significant for protein, oil, C, and N, reflecting that foliar B application alter protein, oil, C, and N. Line had significant effects on protein, oil, K, C, N, and B, showing the significant influence of genotype for seed nutrition. Similar effects of main effects on leaf K, N, and total B (Table 2). The interactions (W×line, B×line, and W×B×line) were also significant, mainly, for protein, oil, and C. W×B×line intercations were also significant for protein, oil, C, and B (Table 1). Main effects of W, B, and line had a significant effects on K and total B, but only W and line had significant on N (Table 2). Interactions of W×B, W×line, and B×line showed significant influence on B (Table 2).

In experiment 1, the mean values for seed nutrition in well-watered plants with no foliar B showed higher accumulation of both protein and seed oil in *N* lines than in *F* lines (Table 3). Seed K, N, and B were higher in *F* lines than in *N* lines, opposing the trend of nutrients in leaves, where K, B, N were higher in *N* lines than *F* lines, and some breeding lines such MD51*ne N* and STV 7A*gl N* performed better than the check for protein and oil, but MD51*ne F* and STV 7A*gl F* had close levels of seed protein and oil with the checks, making them competitive for protein, oil, and some mineral nutrition constituents (Tables 3 and 4). Seed C was higher in *N* lines than in *F* lines in MD51*ne*, though *N* and *F* variants of STV 7A*gl* had similar percentages of C in seeds. Cell wall B (structural B) was higher in *N* lines than in *F* lines, increasing the contribution of cell wall B to the total B to 70% or more (Table 3). Foliar B application under well-watered conditions decreased the contribution of cell wall B to the total B in *N* line compared with *F* lines in MD51*ne*, and the contribution of cell wall B to the total B was lower, up to 58% (Table 3). Foliar B also resulted in higher seed N and B contents in the check and control. Under well-watered conditions, foliar B resulted in higher seed oil in all lines, except in MD51*ne N*, but not in protein. Foliar B resulted in higher leaf B (Table 3), higher seed C in SA 243 and *N* lines only, higher seed N and B in check and control (Table 3).

**Table 3:** Experiment 1: Effect of water (W) and foliar boron application (B) treatments on cottonseed nutrition (protein, oil, K, C, N, B) and nutrients (K, N, B) in leaves (L), and in cell wall B (CellWB), and cell wall B contribution to the total B (CellWB%) in near-isogenic lines expressing fuzzy (F) and fuzzless (N) seed phenotypes.

Line/genotype		Protein^1^	Oil	K	C	N	B	KL	NL	BL	CellWB	CellWB%
				(%)			(mg kg^-1^)	(%)	(%)	(mg kg^-1^)	(mg kg^-1^)	(%)
**SA 243**		30.25	26.00	1.28	50.4	3.70	37.50	1.45	3.55	43.3	30.0	69.3
**PHY 375WRF**		29.25	27.50	1.18	51.0	3.85	41.00	1.73	3.70	51.8	31.8	61.5
**MD 51*ne N***		35.50	36.00	1.30	51.6	3.23	33.75	2.13	4.55	57.5	42.3	73.7
**MD 51*ne F***	W	30.50	30.50	1.33	50.5	3.80	39.75	1.75	4.33	48.5	31.5	65.5
**STV 7A*gl N***		36.75	34.00	1.05	50.5	3.25	32.50	2.13	4.45	55.5	39.5	71.1
**STV 7A*gl F***		32.50	28.25	1.28	50.6	3.93	37.25	1.75	4.13	46.8	27.5	59.3
**LSD**		1.84	1.26	0.10	0.3	0.20	1.57	0.16	0.15	2.1	2.3	4.6
**SA 243**		29.25	33.00	1.28	51.7	4.03	45.00	1.60	3.65	63.5	34.0	53.8
**PHY 375WRF**		34.25	35.25	1.07	50.9	4.15	43.75	1.75	3.93	60.3	40.0	66.8
**MD 51*ne N***		33.50	36.25	1.36	52.4	3.30	34.75	2.33	4.58	67.3	35.3	52.4
**MD 51*ne F***	WB	26.50	33.00	1.33	51.5	3.90	39.75	2.00	3.83	48.5	28.3	58.8
**STV 7A*gl N***		36.00	37.75	1.13	52.4	3.43	34.00	3.33	4.48	68.3	36.3	53.1
**STV7A*gl F***		33.75	35.25	1.25	50.3	3.95	38.25	2.33	3.45	60.0	32.3	53.9
**LSD**		1.43	1.05	0.07	0.3	0.28	1.32	0.13	0.18	2.2	1.6	3.2
**SA 243**		35.50	24.25	0.71	50.6	2.43	31.00	1.25	2.35	30.8	22.8	74.8
**PHY 375WRF**		33.50	23.75	0.72	50.4	2.55	31.50	1.18	2.00	33.8	27.0	79.9
**MD51*ne N***		22.75	23.25	0.69	50.5	2.38	24.25	1.23	2.63	29.8	24.0	81.9
**MD51*ne F***	WS	23.00	22.50	0.68	50.3	2.30	26.25	0.88	2.93	31.3	24.0	77.1
**STV7A*gl N***		37.75	22.50	0.70	50.2	2.20	20.50	1.09	2.45	32.5	28.0	86.7
**STV7A*gl F***		35.25	22.00	0.70	50.4	2.38	19.75	1.03	2.20	30.8	25.0	82.1
**LSD**		1.23	0.75	0.02	0.1	0.20	1.79	0.12	0.21		1.5	6.1
**SA 243**		30.3	23.8	0.838	50.5	3.38	27.3	1.12	2.55	33.8	24.0	71.2
**PHY 375WRF**		30.5	21.8	0.91	50.4	3.2	29.3	3.38	2.23	31.3	25.0	80.8
**MD 51*ne N***	WSB	34.3	30.5	0.685	50.4	3.4	23	1.15	3.08	29.3	23.0	80.4
**MD 51*ne F***		33	30.3	0.823	50.5	3.25	22.5	1.23	2.35	28.8	21.0	74.2
**STV 7A*gl N***		30	29.5	0.623	50.8	3.3	24	1.28	2.38	29.8	27.3	92.4
**STV 7A*gl F***		30.8	28.8	0.748	50.6	3.38	24.5	1.17	2.23	31.8	22.8	73.2
**LSD**		1.46	1.34	0.06	0.2	0.09	1.78	0.88	0.15	1.9	1.2	6.4

^1^Values are means of four replicates. The experiment was repeated twice. Means were separated by Fisher’s protected LSD (0.05).

Abbreviations KL, NL, and BL denote potassium in leaves (KL), nitrogen in leaves (NL), and boron in leaves (BL). Treatments were: well-watered plants without foliar B = W; well-watered plants with foliar B = WB; water-stressed plants without foliar B = WS; water-stressed plants with foliar B = WSB.

Experiment 2 and under well-watered conditions and with no foliar B, seed oil was higher in *N* lines than in *F* lines as in Experiment 1. Also, seed C was higher in *N* line than in *F* line in MD51*ne*, but in STV 7A*gl* lines there were no significant differences (Table 4). Seed K and N were higher in *F* lines than in *N* lines, but B was higher in STV 7A*gl F* line only. Leaf K, N, and B were higher in *N* lines than in *F* lines. A trend of increase in cell wall B was shown in *F* lines than in *N* lines. Except for seed C (where C was higher in STV 7A*gl N* line only) and seed B (where seed B was higher in STV 7A*gl F* line only), similar pattern for seed K, N, and leaf K, N, and B was shown as in Experiment 1under well-watered with no foliar B (Table 4). Under water stress, only STV 7A*gl* showed that seed oil, leaf K and N were higher in *N* line than in *F* line. Cell wall B was higher in *N* lines than in *F* lines under waters stress with foliar B, but no consistent trend was observed under water stress with no foliar B. Foliar B application under water stress conditions resulted in higher seed oil in SA 243 and MD51*ne*, higher in seed N in all lines, including checks, higher seed B in checks, and lower cell wall B in all lines, including checks.

**Table 4:** Experiment 2: Effect of water (W) and foliar boron application (B) treatments on cottonseed nutrition (protein, oil, K, C, N, B) and nutrients (K, N, B) in leaves (L), and in cell wall B (CellWB), and cell wall B contribution to the total B (CellWB%) in near-isogenic lines expressing fuzzy (F) and fuzzless (N) seed phenotypes.

Line/genotype		Protein^1^	Oil	K	C	N	B	KL	NL	BL	CellWB	CellWB%
				(%)			(mg kg^-1^)	(%)	(%)	(mg kg^-1^)	(mg kg^-1^)	(%)
**SA 243**		25.0	25.8	1.17	50.8	3.65	36.0	1.33	3.53	42.5	29.5	69.8
**PHY 375WRF**		28.3	31.3	1.10	50.5	3.98	42.8	1.93	3.85	45.3	30.8	67.9
**MD 51*ne N***		24.5	30.3	0.92	51.3	3.20	37.8	1.73	4.20	55.5	40.0	72.1
**MD 51*ne F***	W	28.8	25.0	1.15	50.5	3.93	36.5	1.48	3.78	50.5	33.5	66.5
**STV 7A*gl N***		23.8	32.3	1.00	52.0	3.35	36.8	2.83	4.13	55.3	33.5	60.7
**STV 7A*gl F***		26.5	26.5	1.20	52.0	4.45	42.3	1.93	3.53	45.5	24.5	54.7
**LSD**		1.2	1.2	0.05	0.3	0.19	1.7	0.17	0.23	1.5	1.5	4.1
**SA 243**		29.3	28.5	1.01	50.8	3.88	40.5	1.80	4.15	53.8	30.8	57.9
**PHY 375WRF**		35.8	30.5	1.20	51.0	4.58	39.5	2.40	4.00	58.3	33.8	58.5
**MD 51*ne N***		29.8	31.0	1.10	52.8	3.75	41.5	2.70	4.00	58.5	31.0	53.0
**MD 51*ne F***	WB	28.3	27.8	1.13	52.7	4.60	41.3	2.38	4.05	55.3	29.3	52.9
**STV 7A*gl N***		34.0	33.5	1.07	52.2	4.28	33.3	2.83	4.58	60.5	33.0	54.9
**STV7A*gl F***		32.8	29.0	1.20	50.3	4.83	36.8	2.48	3.53	57.0	29.3	51.4
**LSD**		1.4	1.1	0.05	0.3	0.18	1.7	0.17	0.22	0.2	1.8	4.1
**SA 243**		30.3	24.8	0.88	50.5	2.68	27.75	1.33	2.93	31.25		87.3
**PHY 375WRF**		32.5	25.5	0.81	50.4	2.60	28.6	1.10	3.05	30.5	23.0	77.6
**MD51*ne N***		24.0	23.0	0.88	50.5	2.68	27.8	1.33	2.93	31.3	26.8	87.3
**MD51*ne F***	WS	24.5	22.8	0.80	50.5	2.45	29.3	1.28	3.38	30.3	25.3	84.2
**STV7A*gl N***		28.3	26.0	0.85	50.3	3.35	29.8	1.60	3.93	32.3	26.5	82.4
**STV7A*gl F***		32.3	23.0	0.81	50.2	3.28	28.3	1.28	3.05	33.5	29.5	88.3
**LSD**		2.7	2.7	0.04	0.2	0.24	2.0	0.12	0.21	1.7	1.1	6.1
**SA 243**		27.3	27.5	0.81	50.5	3.475	32.0	1.20	2.88	27.8	22.3	81.5
**PHY 375WRF**		33.5	24.3	0.85	50.3	3.735	35.1	1.58	3.30	32.8	23.8	70.8
**MD 51*ne N***		30.0	27.0	0.75	50.4	3.95	22.8	1.35	3.30	29.8	24.8	76.2
**MD 51*ne F***	WSB	29.0	27.8	0.76	50.2	4.325	24.8	1.23	3.43	30.8	23.5	70.2
**STV 7A*gl N***		33.8	26.4	0.85	50.3	3.575	30.3	1.30	3.68	33.5	23.0	77.7
**STV 7A*gl F***		32.8	24.3	0.83	50.2	3.733	29.8	1.18	3.23	33.5	22.5	74.4
**LSD**		1.6	0.8	0.05	0.1	0.19	1.4	0.10	0.14	1.4	1.0	0.6

^1^Values are means of four replicates. Means were separated by Fisher’s protected LSD (0.05).

Abbreviations KL, NL, and BL denote potassium in leaves (KL), nitrogen in leaves (NL), and boron in leaves (BL). Treatments were: well-watered plants without foliar B = W; well-watered plants with foliar B = WB; water-stressed plants without foliar B = WS; water-stressed plants with foliar B = WSB.

The higher seed oil in *N* lines compared with *F* lines could be due the inherited genetic differences in carbon metabolism (carbon is a source of oil) and storage availability of sugars (source of energy). Since fuzzy-linted lines produced more lint than fuzzless-linted lines [28], it is reasonable to assume that more energy was used to produce lint in *F* lines and less energy in *N* lines, converting the stored energy to oil, and probably to sugars. Further research is needed to investigate the concentrations of seed sugars and sugar types (sucrose, raffinose, and stachyose) involved in oil distribution. The higher oil concentration that resulted from foliar B application could be due to the indirect effects of B on carbon and sugar metabolism through carbohydrate transport and fatty acid and oil synthesis. This could be supported by the fact that foliar B application resulted in higher C in *N* lines than in *F* lines, indicating a possible higher energy storage and use for fatty acid synthesis. For example, in experiment 1, C% in seeds was 51.60 vs. 50.60 with LSD = 0.3; and 52.38 vs. 51.50 with LSD = 0.3, respectively in *N* lines vs. *F* lines. Same trend was observed in Experiment 2, supporting the previous observation (Tables 3 and 4). Under water stress this trend was not observed may be due to drought stress (low soil moisture) effects on B uptake and translocation. In the current experiment, it is clear that the accumulation of B in seeds and leaves under water stress was significantly reduced compared with under well-watered conditions (Table 3 and Table 4). Previous research showed that in a 2-year field experiment on a B-deficient calcareous soil where soil-applied B on cotton was applied at rates of 0.0, 1.0, 1.5, 2.0, 2.5, and 3.0 kg B ha^−1^, as borax, and it was found that B soil application resulted in an increase in seed oil content (P <0.05) and seed protein content (P < 0.05) in both years [16]. They concluded that cotton growth, productivity, and nutritional value of cotton seeds were cost-effectively improved by optimizing B nutrition. They added that fertilizer B requirement was 1.0 kg B ha^−1^, soil B critical range was 0.5–0.6 mg kg^−1^ soil, and critical B requirements for leaves varied with leaf and plant ages, ranging from 38.0 mg kg^−1^ to 55.0 mg kg^−1^ [16].

There is no previous research available on the effects of B on fuzzy and fuzzless cottonseed lines. The research available in this area was done on conventional varieties in different species, including cotton, and the results showed that foliar B application resulted in higher oleic fatty acids [38], higher seed protein and higher oil [16, 43], or lower oil [38]. The effect of B on seed nutrition was previously explained as indirect effects of B on nitrogen and carbon metabolism, and its interaction with other minerals. For example, the effect of B nutrition on carbon and nitrogen metabolism and its indirect involvement with these processes [28, 44–46] was previously reported. In the current experiment, the higher accumulation of K, N, and B in seeds in leaves of *N* lines than in *F* lines could be due to limited mobility and translocation of these nutrients from leaves to seed in *N* lines. The mechanism of limited translocation of nutrients from leaves to seed occurred even when foliar B was applied, and this could be due to limited B mobility through the phloem within the cotton plant [45, 47]. The lack of translocation in cotton could be explained by lower nutrient demand by *N* lines, as *N* lines produce lower lint yield [29]. Although B mobility in cotton is limited and genetically controlled, possibly due to, partially, lack of enough sugar alcohol responsible for phloem mobility of B [47], the limited uptake of B by roots and transport of B from leaves to seed could be initiated by signals, transporters, or protein channels [48–51]. The higher B accumulation under well-watered conditions is due to both foliar B and B uptake by roots from soil as B is passively taken up through passive mass flow of water [52] due to high soil moisture due to watering. Beside the passive mass flow, other processes for B transport and mobility were also reported and involved polyols (sugar alcohol such as mannitol and sorbitol) [47, 52], aquaporins (water channels) [48, 53, 54], and their contribution to water uptake under drought [55,56], and the recent discovery of B transporters [50] and active B uptake [57, 58]. The positive response of some nutrients such as C and N in seeds or N and K in leaves to foliar B application in some lines could be due to the interactive relationships between B and these nutrients and positive and negative correlation between cation and anion nutrients were previously reported [44]. The response of these nutrients to foliar B in some lines only could be due to genotype differences due to nutrients requirements [28].

The lower concentration of cell wall B under well-watered conditions and the higher cell wall B concentration under water-stressed conditions can be due to the fact that B has mainly a structural role, i.e., its integration in cell wall structure and function [59–61] and cell membrane [44] is the main priority. Therefore, when there is enough B available in the cell, a small amount of B will be integrated in the cell walls. However, when the concentration of B is smaller in the cell, plants will integrate B to cell walls as cell walls synthesis is the first priority of the plant to protect the cell from biotic or biotic stresses, including drought/water stress. This process could be stimulated through signals or other unknown mechanisms. The higher protein under water stress/drought conditions was also found by other researchers [3, 62] and was reported to be due to the genetic control of the inverse relationship between protein and oil or lower seed size and smaller seed biomass under drought conditions.

## Conclusions

The current research demonstrated that fuzzless cottonseed accumulated higher oil content, but no consistent trend for seed protein was noticed, indicated that the seed fuzziness trait may be involved in oil and protein accumulation in seeds. Nutrients N, K, and B were higher in fuzzy seeds than fuzzless seeds, but the concentrations of these nutrients in fuzzless seed were higher in leaves than in seeds, opposing the trend in fuzzy seeds, may be due to limited translocation of these nutrients from leaves to seeds due to unknown mechanisms. The positive response of K, N, and B in leaves and seeds to foliar B application under well-watered or water stress conditions dependent on genotype, allowing for possible selection for more responsive genotypes to B application. Water-stress altered both amount and trend of seed nutrients in seeds and leaves. Foliar application of B under water stress may not increase B in seeds, may be due to limited movement of B from leaves to seed due to water stress, may indicate that the beneficial effects of foliar B under water is limited. Under water stress, the contribution of cell wall B to the total B exceeded 90%, supporting the structural role of B in plants. Foliar B application decreased the contribution of cell wall B to the total B, may be due to the non-limiting availability of B in the cell. Further research at the biochemical and molecular levels is needed to understand mechanisms controlling the distribution of seed nutrition. To evaluate the stability of the trait and its effects on seed nutrition, more lines under different environments need to be investigated.
